# Central Nervous System Metastasis in Neuroblastoma: From Three Decades Clinical Experience to New Considerations in the Immunotherapy Era

**DOI:** 10.3390/cancers14246249

**Published:** 2022-12-19

**Authors:** Angela Mastronuzzi, Giovanna Stefania Colafati, Andrea Carai, Maria D’Egidio, Francesco Fabozzi, Francesca Del Bufalo, Maria Felicia Villani, Giada Del Baldo, Sabina Vennarini, Costanza Canino, Angela Di Giannatale, Paolo Tomà, Maria Carmen Garganese, Maria Antonietta De Ioris

**Affiliations:** 1 Department of Hematology/Oncology, and Cell and Gene Therapy, Bambino Gesù Children’s Hospital, IRCCS, 00165 Rome, Italy; 2 Faculty of Medicine and Surgery, Saint Camillus International University of Health Sciences, 00131 Rome, Italy; 3 Department of Imaging, Bambino Gesù Children’s Hospital, IRCCS, 00146 Rome, Italy; 4 Neurosurgery Unit, Department of Neurosciences, Bambino Gesù Children’s Hospital, IRCCS, 00165 Rome, Italy; 5 Pediatric Radiotherapy Unit, Fondazione IRCCS Istituto Nazionale dei Tumori, 20133 Milan, Italy

**Keywords:** neuroblastoma, central nervous system metastasis, central nervous system relapse

## Abstract

**Simple Summary:**

Central nervous system (CNS) metastatic spread in neuroblastoma (NB) is rare and occurs more often at relapse/progression. In this retrospective study, we reviewed the CNS imaging of all the patients treated at the Bambino Gesù Children Hospital over a 25-year period. CNS metastasis in NB is confirmed to be rare, occurring in 4.7% of patients, and associated with advanced disease and bone skull involvement. In the last decade, the involvement of CNS at relapse has been observed more frequently, supporting the rising concern of the impact of immunotherapy in the pattern of relapse in high risk (HR) NB. Further studies are needed to confirm a higher CNS relapse risk in the immunotherapy era as well as the need for including CNS imaging in follow-up.

**Abstract:**

Central nervous system (CNS) metastatic spread in neuroblastoma (NB) is rare and occurs more often at relapse/progression. We report on CNS involvement in high risk (HR) NB over 25 years. For this retrospective study, we reviewed the CNS imaging of all the patients treated at Bambino Gesù Children Hospital from 1 July 1996 to 30 June 2022. A total of 128 patients with HR NB were diagnosed over 26 years. Out of 128 patients, CNS metastatic spread occurred in 6 patients: 3 patients presented a metastatic spread at diagnosis, while in 3 patients, CNS was involved at relapse. Overall, the rate of occurrence of CNS spread is 4.7% with the same distribution at diagnosis and at relapse, namely 2.3%. Interestingly, CNS spread at diagnosis was observed only before 2012, whereas CNS was observed at relapse only after 2012, in the immunotherapy era. CNS metastases presented similar imaging features at diagnosis and at relapse, with a peculiar hemorrhagic aspect and mainly hemispheric localization in patients with bone skull involvement at the time of diagnosis. The outcome is dismal, and 3 out of 6 patients died for progressive disease.

## 1. Introduction

Neuroblastoma (NB) is the most common extracranial solid tumor in children, accounting for 8–10% of all pediatric cancers and about 15% of cancer-related deaths [1,2]. NB is marked by a heterogeneous clinical behavior, ranging from spontaneous regression in infants to an aggressive disease in patients over 18 months of age with either metastatic disease or MYCN amplification, defined therefore as high-risk (HR) [3]. Secondary lesions are present in more than 50% of patients, mainly located in the bone, bone marrow, and liver [4,5,6,7,8]. Metastatic involvement of the skull, orbit, or skull base is common [6,9,10], while central nervous system (CNS) metastases are rare, with an overall estimated prevalence of 1.7–11.7% [11,12,13,14,15,16,17,18]. CNS involvement may be present at the time of presentation, but in most cases, occurs at progression/relapse [6]. According to the International Neuroblastoma Risk Group classification, HR NBs are treated with an intensive strategy based on conventional and high-dose chemotherapy followed by autologous hematopoietic stem cell rescue, surgery, radiotherapy, and immunotherapy. The survival improved in the last decade with the introduction of immunotherapy. Indeed, the event-free survival (EFS) rate reaches about 50% [19,20,21]. In recent years, thanks to the implementation of such aggressive multimodal treatment protocols, the life expectancy of pediatric patients with HR NB has improved; however, CNS metastatic lesions seem to be more frequently observed [15,19,22,23,24,25,26,27].

In the present study, we reviewed the cases of HR-NB diagnosed and treated at our institution over 26 years and performed an accurate radiologic revision of all the CNS imaging, to assess the occurrence and the characteristics of CNS metastatic spread, evaluating the impact of the introduction of immunotherapy.

## 2. Materials and Methods

Data were obtained from a retrospective review of patients with HR NB treated at the Department of Hematology/Oncology, Cell and Gene Therapy at Bambino Gesù Children Hospital from 1 July 1996 to 30 June 2022. All patients were treated according to the NB HR strategy with conventional and high-dose chemotherapy followed by autologous hematopoietic stem cell rescue, surgery, radiotherapy, and immunotherapy after 2011. 

All NB patients with MYCN amplified tumor or with metastatic tumor older than 12 months are considered HR; only recently, the patients with metastatic tumor younger than 18 months of age at diagnosis without MYCN amplification and without segmental chromosomal abnormalities (SCA) were included in the intermediate-risk group.

Collected data included sex, age, tumor localization at diagnosis, tumor staging, metastatic status at diagnosis, histology, MYCN status, treatment details, surgery, radiological evaluations and clinical outcome. 

For all patients with CNS spread at diagnosis or at progression/relapse, a radiology information system query was performed to recall the computed tomography (CT), magnetic resonance imaging (MRI), and ^123^I- metaiodobenzylguanidine (MIBG) scan examinations of the brain and spine; the imaging findings were reviewed, reaching a consensus image interpretation among a senior pediatric neuroradiologist (GSC) and a junior resident radiologist (MDE) and by a nuclear medicine specialist (MCG). Imaging findings were reviewed on picture archiving and communication systems (PACS). Data of CNS involvement assessed with CT/MR imaging and ^123^I-MIBG scan included type of CNS involvement; anatomical location of intracranial metastases (IM); number of IM; maximum diameter of IM; vasogenic edema associated with IM; density of IM on CT; contrast enhancement of IM; presence or absence of hemorrhage, cysts, and necrosis; and ^123^I-MIBG uptake.

All investigations were conducted according to principles expressed in the Declaration of Helsinki, and the study was approved by the Internal Review Board (IRB) of the Bambino Gesù Children’s Hospital.

OS was defined as the time from diagnosis to death, and survival after the first progression (SFP) was defined as the time from first progression of the disease to death. Comparison tests included Fisher’s exact test and Student’s T-test distribution. OS and SFP were calculated using the Kaplan–Meier method. Survival curves were compared with log-rank tests. All comparison tests were two-sided and considered significant at the 5% level. Patient data were analyzed using IBM SPSS Statistics Version 22 for Windows.

## 3. Results

A total of 128 patients with HR NB were diagnosed over 26 years; out of them, a CNS metastatic spread was observed in 6 patients. In detail, 3 patients presented a metastatic disease at diagnosis, and 3 patients at relapse. 

Table 1 summarizes the clinical characteristics of patients with CNS metastatic spread. 

The overall occurrence rate of CNS spread is 4.7% (6/128) and is superimposable at diagnosis and at relapse, being 2.3% in both cases. Intriguingly, the rate of occurrence at diagnosis and relapse was different, stratifying the patients in two different timeframes, according to the introduction of immunotherapy in 2012. In particular, in the first period, from 1996 to 2011, we observed a CNS involvement rate at diagnosis of 4.4% (3/68) and no cases of CNS relapse. In the more recent patient cohort, from 2012 to 2022, we did not observe any CNS metastasis at diagnosis but an occurrence rate of CNS relapse of 5% (3/60). Considering an older period (until 2011) and a more recent period (from 2012), the 5-year event-free survival (EFS) is 30.2% (CI 19.6–41.4%) and 50% (CI 30.4–64%) (*p* < 0.05), respectively, while the 5-year overall survival (OS) is 38.3% (CI 26.7–49.8%) and 61.4% (CI 44.7–76.5%) (*p* < 0.05), respectively. Considering the 3 patients with CNS metastasis at diagnosis, only 1 is alive at 121 months, while out of 3 patients with CNS relapse, 2 are still alive, respectively, at 114 and 30 months from diagnosis. In Table 2, the imaging features of patients with metastatic CNS spread at diagnosis or relapse are summarized.

All patients with CNS metastatic spread, both at diagnosis and at relapse, presented bone marrow and bone involvement at the time of diagnosis; of notice, skull involvement was evident in all patients with CNS spread. Symptoms were observed in 3 out 6 patients; headache and motor impairment were the main symptoms.

The imaging findings were reviewed for all patients; in two patients, few imaging findings were too outdated. Among the 6 patients with intracranial metastasis (IM), 4 patients had intraparenchymal supratentorial lesions, 1 had an intra-parenchymal infratentorial lesion, and 1 presented with multiple brain and spine leptomeningeal nodular lesions. Of notice, 2 patients (namely patient 2 and 3) with CNS metastases were referred to our Institution after diagnosis and removal of the CNS lesions, and thus baseline imaging is not available. The ^123^I-MIBG scans were available in 5 patients; for 2 patients, both with IM at diagnosis, the ^123^I-MIBG scan was performed after radical surgery while between the 3 patients with IM, at relapse ^123^I-MIBG scans were positive in 2 patients. 

**Figure 1:**
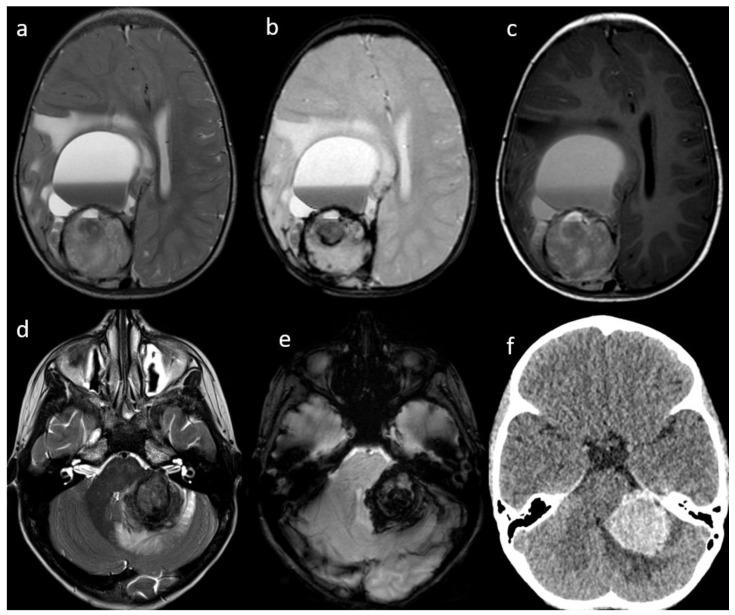
Patient #1 (**a**–**c**) and Patient #3 (**c**–**e**) imaging features. Axial T2w images of Patient #1 (**a**) and Patient #3 (**d**) shows a single bulky parenchymal lesion associated with mild surrounding vasogenic edema. Susceptibility-weighted imaging sequences in Patient #1 (**b**) and Patient #3 (**e**) show blooming within each lesion consistent with hemorrhage. Axial T1w image of Patient #1 (**c**) shows a solid-cystic appearance of the lesion with multiloculated blood-fluid levels in the most anterior portion and a solid hemorrhagic posterior component. Axial non contrast brain computed tomography (CT) of Patient #3 (**f**) shows lesion hyperdensity consistent with hemorrhage.

**Figure 2:**
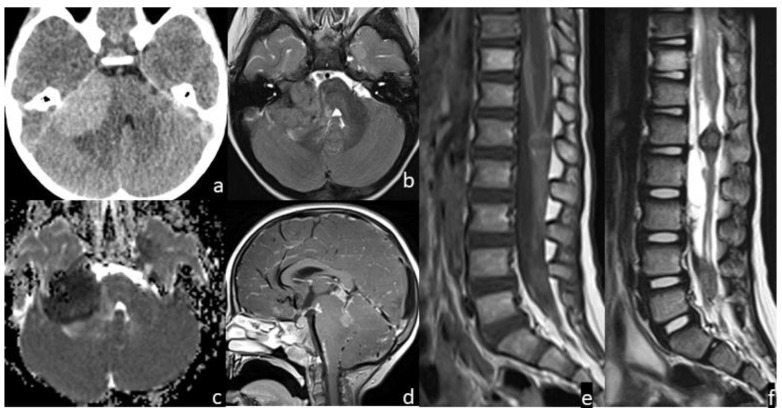
Patient #2 imaging features. Axial non contrast brain computed tomography (CT) (**a**) showed a bulky central nervous system (CNS) hyperdense lesion within the right cerebellopontine angle, with inhomogeneous signal in axial T2w image (**b**), but with restricted diffusion in diffusion weighted imaging (DWI) (**c**). Post-contrast sagittal T1w image of the brain (**d**) and post-contrast sagittal T1w (**e**) and T2w (**f**) images of the spine showed numerous other brain and spine leptomeningeal lesions. MRI of the spine demonstrated thickened leptomeninges with multiple extra medullary enhancing nodular lesions (**e**); the nodular enhancing foci within the conus and along the nerve roots of the cauda equine (**f**) had an inhomogeneous low signal intensity on sagittal T2w images indicating a hemorrhagic component within them.

**Figure 3:**
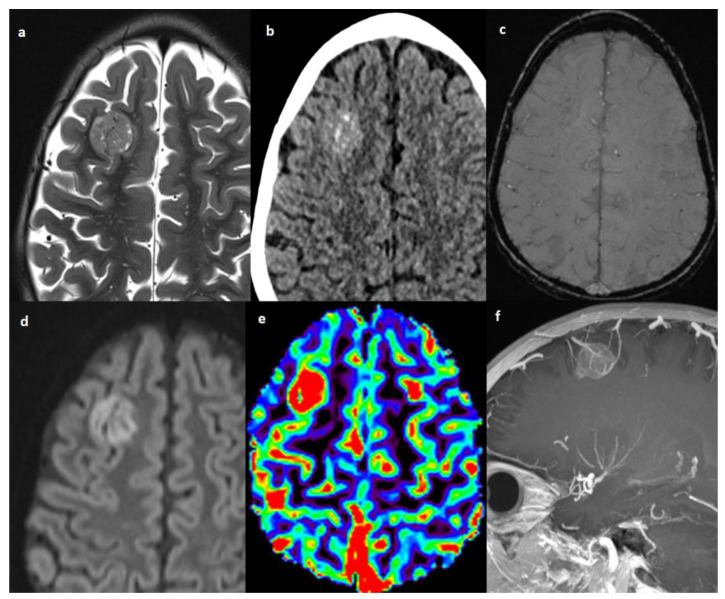
Patient #4 imaging features. Axial T2w image (**a**) shows a single lesion in the right frontal lobe, hyperdense on axial non contrast brain computed tomography (CT) (**b**), without hemorrhagic components in susceptibility weighted imaging (SWI) (**c**), but with restricted diffusion in diffusion weighted imaging (DWI) (**d**). The cerebral blood volume map from dynamic susceptibility contrast (DSC) perfusion images (**e**) shows elevated relative cerebral blood volume (rCBV) within the lesion consistent with angiogenesis/vascular proliferation. Post-contrast sagittal T1w image with maximum intensity projection (MIP) reconstruction (**f**) shows tortuous vessels within and around the lesion.

The IM were all surrounded by only a small amount of vasogenic edema, with the most edemigen lesions being those characterized by a hemorrhagic appearance; however, none had a large volume of peritumoral brain edema. Hemorrhage was seen in 4 patients with brain parenchyma lesions and in the patient with the diffuse brain and spine leptomeningeal involvement; one of the two hemorrhagic parenchymal lesions had a solid-cystic appearance with multiloculated blood-fluid levels (Figure 1 and Figure 2). The one non-hemorrhagic brain parenchyma localization and the greatest intracranial lesion in the leptomeningeal CNS involvement presented with intralesional and perilesional prominent blood vessels. Dynamic susceptibility contrast (DSC) MR perfusion was performed in the patient with the non-hemorrhagic brain parenchyma localization and it demonstrated an abnormally elevated perfusion in the tumor, according with the presence of prominent intra- and perilesional vessels (Figure 3). All IM appeared hyperdense on CT scans and moderately hypointense in T2 weighted images (T2 WIs). Diffusion-weighted imaging (DWI) was significantly distorted in 2 patients for hemorrhage; on the other hand, restricted diffusion was present in the patient with the non-hemorrhagic brain parenchyma lesion and in the greater nodular leptomeningeal intracranial localizations. Contrast enhancement (CE) was present in all patients with IM, even if it was of difficult evaluation in hemorrhagic lesions. 

All patients received at diagnosis the treatment according to NB HR strategy as detailed above. The only survivor patient with CNS involvement at diagnosis received focal radiation at 21 Gy on CNS lesion after surgery. Among CNS relapses, all patients received a second-line chemotherapy, surgery on primary tumors and craniospinal irradiation (CSI) at 21 Gy. In the setting of relapsed patients, a combination of temozolomide and irinotecan was used (details of treatment at diagnosis and at relapse in Table 1).

## 4. Discussion

We report on CNS involvement in HR NB over a long period. In our population, the overall incidence of brain metastases was 4.7% (6/128), consistent as reported in the literature [15,16,17,18] and very close to the incidence of 5.3% reported in a French retrospective analysis, which considered 434 children with stage 4 NB diagnosed during a long time period (1985 and 2000) [11]. In this series, we observed an occurrence rate at diagnosis of 4.4% (3/68) and no CNS spread at relapse in an older period before 2012, while in the last 10 years, we did not observe any CNS metastasis at diagnosis and an occurrence rate of CNS relapse of 5% (3/60). Our data suggest, therefore, a higher occurrence of CNS relapses in the last decade, and this result may support the rising concern on the impact of new therapeutic strategies, such as immunotherapy, on the pattern of relapse in HR NB patients. Indeed, data reported so far in the literature support the idea of an increasing rate of CNS involvement over time as a result of both a better control of the disease in the other metastatic sites and the impossibility of anti-disialoganglioside (GD2) antibodies to cross the blood–brain barrier (BBB) [11,12,15,28]. In our series, the 5-year EFS improves from 30 to 50% in the two different periods, supporting the idea of different patterns of relapse in recent years. Indeed, anti-GD2 antibodies do not penetrate the BBB, thus potentially allowing the CNS to represent a “sanctuary site” for NB cells, resulting in a higher proportion of CNS recurrences in children undergoing immunotherapy [15,28,29,30]. Among 127 patients with stage 4 NB diagnosed at Memorial Sloan Kettering (MSK), 8 patients (6%) developed CNS relapses, with a tendency toward a higher number of CNS relapses among patients previously treated with immunotherapy and no high dose chemotherapy (HDC), in comparison with patients that received HDC and no immunotherapy (1/60) [15]. In the German experience, CNS relapses were reported to occur in 49 out of 451 patients (11%) treated with HDC as part of the first-line treatment, with some of these patients receiving anti-GD2 antibodies as part of the maintenance therapy [28]. These data were not confirmed in the prospective trial of the European International Society of Pediatric Oncology Neuroblastoma Group, where the trend toward a higher proportion of CNS recurrences over time was not observed, and the risk of CNS recurrence was found to be linked to both patient and disease characteristics, with no impact of neither high dose chemotherapy nor immunotherapy [31]. In the HR-NBL1/SIOPEN trial experience female sex, MYCN amplification, hepatic, and >1 metastatic system/compartment involvement, instead, were identified as significant risk factors for CNS relapse [31]. However, in our population, the female:male ratio is 1:2, only one patient with CNS at diagnosis had liver metastasis and only one was affected by tumor-carrying MYCN amplification; on the other hand, all patients had diffuse metastatic disease with skull involvement at diagnosis. According to our experiences, an impressive skull involvement at diagnosis seems associated with CNS spread both at diagnosis and at relapse. This finding is not confirmed by large series as in the HR-NBL1/SIOPEN trial. Of note, the skull involvement was not evaluated as a different variable from skeleton metastasis, taking into account different findings due to our limited number of patients. Moreover, HR-NBL1/SIOPEN trial may be affected by underreporting, since CNS imaging was not requested in patients without MIBG-avid skull metastases, and thus it may have not been performed in multi-metastatic patients without neurological symptoms.

Main published cohorts reporting the occurrence of CNS metastasis are summarized in Table 3.

The CNS involvement may be asymptomatic, but rapid and fatal symptoms were reported and may represent a matter to debate for routine CNS imaging screening in HR NB [32]. Moreover, not only CNS involvement can be asymptomatic, but even the ^123^I-MIBG may not reveal IM. The CNS involvement at ^123^I-MIBG scintigraphy may be negative in more than half of the patients in some series [11]. Not only MIBG does not cross the blood−brain barrier (BBB) [33], but it is possible to speculate that peritumoral brain edema in metastases and hemorrhage can contribute to false negative MIBG scans. Two out of three relapsed patients had positive MIBG scans at the time of CNS recurrence with definite visualization of the lesion by MIBG uptake, whereas the other patient with a major amount of vasogenic edema surrounding the hemorrhagic lesion had negative results.

A greater emphasis should be placed on performing regular neurological monitoring and obtaining warranted imaging studies also because the increased levels of urinary catecholamine metabolites as clinical biomarkers for the detection of metastasizing NB may not be a reliable indicator of CNS disease [11] as it was shown in our population. None of the CNS relapse provided a slight increase in urine metabolites, raising the recurrence suspicion, confirming that CT scan and especially contrast-enhanced MRI imaging studies are the golden standard for CNS metastases diagnosis in NB [34]. 

CNS metastases at diagnosis or relapse shared the same imaging characteristics; peculiar features of CNS spread in NB seem to be the hemorrhagic aspect, the little degree of associated vasogenic edema and a single enhancing parenchymal supratentorial lesion which is hyperdense on CT and moderately hypointense in T2 WIs. These imaging characteristics are consistent with those previously reported in the literature [11,15,35]. Therefore, brain or spine hemorrhage lesions in NB patients should be considered suspicious for metastatic disease, and appropriate imaging should be performed. Restricted diffusion was detected, accounting for the high cellularity of this tumor [36]; however, diffusion could be difficult to evaluate in hemorrhagic lesions. High cellularity also justifies the characteristic CT hyper-density and T2 WIs hypo-intensity of IM, peculiar unvaried imaging findings of NB CNS metastases, even in non-hemorrhagic lesions. The only patient who performed DSC MR perfusion imaging revealed tumor hyper-perfusion, consistent as was previously reported [36]. Cerebral blood volume (CBV) is related to microvascular proliferation in tumors and is a surrogate marker of angiogenesis [37]; moreover, high CBV was an expected finding because of the evidence of increased intravascular fluid volume due to prominent intra- and perilesional vessels in the same patient. 

It has been reported that younger age and bone marrow involvement at initial diagnosis were associated with the subsequent development of brain metastasis in metastatic NB [38]; indeed, all patients with IM in our population had skull metastases at the time of diagnosis. The contamination of CNS by circulating tumor cells in the blood at the time of diagnosis as the CNS involvement by continuity with skull bone infiltration was considered a possible mechanism of CNS metastatic diffusion. Several mechanisms of tumor cell invasion have been considered to date, including penetration starting in the affected meninges, topographically associated with involved cranial bones, active penetration through the meninges and dissemination through CSF, epidural microscopic tumors cells seeding in the craniospinal axis during diagnostic procedures in patients with known bone marrow disease, and hematogenic involvement [39]. 

Routine CNS screening could improve the rate of early diagnosis and prognosis of NB children with IM [10], who have a dismal prognosis as shown in the HR-NBL1/SIOPEN trial experience [31]. Only further and prospective studies can confirm the utility of CT/MRI scan in routine follow-up screening and the real impact of early diagnosis on survival. 

The survival rate is dismal considering both CNS metastasis at relapse or diagnosis. Out of the 6 patients with IM, 3 are still alive: 1 patient with CNS involvement at diagnosis after 121 months; and 2 patients with CNS relapses at 114 and 30 months. The survival time after the diagnosis of CNS involvement seems longer than reported in the Polish [32] and French [11] cohorts. 

Chemotherapy, CSI, and surgery-based comprehensive treatment can prolong the survival time [6]. In the HR-NBL1/SIOPEN trial experience [31], 3/17 of patients with isolated CNS relapses who were treated with complete surgery, CSI, and chemotherapy—mainly the temozolomide-containing regimen—were long-term survivors. Four out of 10 children with CNS relapse treated between 1978 and 1989 at the St. Jude Children’s Research Hospital received as well CSI, surgery, and chemotherapy, and two of them were alive and free of disease at 50 and 62 months after CNS relapse [40]. In a Memorial Sloan Kettering (MSK) retrospective analysis of 29 patients with CNS relapses treated between 1987 and 2007, none treated before 2003 with focal radiotherapy survived, although 12 of 16 patients were treated with surgical resection, craniospinal irradiation, followed by chemotherapy (irinotecan, temozolomide, and carboplatin) and intrathecal radio-iodinated monoclonal antibodies (3F8 or 8H9), were alive without CNS disease with a median of 28 months of follow-up [15,41]. Of note, the results published recently by the MSK seem very promising [42]. In this study, 94 patients with CNS relapse diagnosed between 2003 and 2019 were treated with CSI 18 Gy or 21 Gy, and intrathecal immunotherapy with 131I-8H9 or 131I-3F8, achieving a 5-year OS over 40%, with more than 65% of the patients remaining CNS-disease free after 5 years. Furthermore, reducing the CSI dose from 21 Gy to 18 Gy resulted in similar CNS tumor control [42]. In our case series, the only surviving patient with CNS involvement at diagnosis was given only focal RT at 21 Gy on the CNS lesion (the patient was younger than 3 years old of age). Among patients with CNS relapse, all underwent CSI as advocated in recent years. 

The CNS metastatic spread in NB is confirmed to be rare and almost associated with an advanced disease and bone skull involvement accounting for a contiguous diffusion mechanism at diagnosis. CNS relapse seems to be a pattern of spread more common in recent years after the introduction of immunotherapy. The outcome of HR NB has changed in the last two decades with an improvement mainly due to immunotherapy; in our experiences, CNS metastatic spread has grown with the increasing outcome as confirmed by EFS/OS improvement and the higher CNS relapse incidence in the last decade. The higher incidence may be the result of better control of different metastatic sites and lack of the GD2 antibodies BBB penetrance. Considering this hypothesis, cell immunotherapy with anti-GD2 CAR-T cells may overcome this limitation of GD2 antibodies [43]. The outcome is dismal as reported in most series. Encouraging data—mainly due to the MSK experiences—support surgery, CSI and intrathecal immunotherapy. 

Further analysis, in large series treated with immunotherapy, may confirm an increased risk of CNS relapses in the immunotherapy era. Moreover, the need for CNS imaging during the follow-up of HR NB is a matter to debate; the CNS imaging is mandatory in symptomatic patients, and hemorrhagic CNS lesions should be considered almost suggestive for relapse. According to our experience, CNS active surveillance may be considered in patients most at risk, such as those with skull metastases at baseline and in patients with CNS involvement at diagnosis. Moreover, any neurological symptoms in the follow-up period should be considered with caution, and CNS imaging may be performed. In our experience, the gold-standard for NS diagnosis is brain MRI.

## 5. Conclusions

Our series shows that patients affected by CNS metastasis of NB experience a poor prognosis. We noted an increased incidence of CNS relapse in patients treated with anti-GD2 antibodies, mirroring their scarce BBB penetrance, calling for a newer immunotherapeutic approach that may overcome this limitation. In addition, further studies must be warranted to investigate if and how to perform CNS surveillance during the follow-up of patients affected by HR NB.

## Figures and Tables

**Table 1:** Patient characteristics.

Patient	Sex	Age at dg	Primary Tumor	Metastasis	Skull Involvement at dg	MYCN Status	Urinary Catecholamines at dg/Relapse	Relapse	CNS Relapse	Time to CNS Relapse	Treatment at Relapse	Outcome
1	F	18	Adrenal gland	Bone/marrow, liver, CNS	yes	Not amplified	Positive	No	no	N/A	N/A	Alive, 121 mo
2	F	60	Adrenal gland	Bone/marrow, CNS	yes	Not amplified	Positive	yes, 21 mo	no	N/A	N/A	Died, 45 mo
3	M	31	Adrenal gland	Bone/marrow, CNS	yes	Not amplified	Positive	yes, 37 mo	no	N/A	N/A	Died, 77 mo
4	F	19	Adrenal gland	Bone/marrow, Lymphonode	yes	Amplified	Positive/negative	yes, 27 mo	yes, first relapse	27 mo	Surgery, chemotherapy, CSI	Alive, 114 mo
5	M	86	Adrenal gland	Bone/marrow	yes	MYCN gain	Positive/negative	yes, 25 mo	yes, first relapse	25 mo	Surgery, chemotherapy, CSI	Died, 47 mo
6	M	18	Adrenal gland	No (bone/marrow at first relapse)	No, yes at first relapse	MYCN gain	Positive/negative	yes, 12 mo	yes, second relapse	24 mo	Surgery, chemotherapy, CSI	Alive, 30 mo

LEGENDS: F, female; M, male; dg, diagnosis; CNS, central nervous system; mo, months; N/A, not available.

**Table 2:** Imaging features.

Patient	Type of CNS Involvement	Location	Number of Lesions	Size (mm)	Imaging Modality	Enhancement	Density	Cyst, Necrosis	Edema	Hemorrhage	Diffusion	Perfusion	^123^I MIBG Scan
1	brain parenchyma	right cerebral hemisphere	1	90 × 60 × 50	CT MRI	yes	Yes	yes	yes	yes	N/A	N/A	performed after surgery
4	diffuse leptomeningeal	supratentorial and infratentorial brain regions; pineal and sellar regions; spine and cauda	>1	N/A	CT MRI	yes	High	no	yes	yes	restricted	N/A	positive
5	brain parenchyma	left cerebellar hemisphere/middle cerebellar peduncle	1	30 × 30 × 36	CT MRI	yes	High	no	yes	yes	N/A	N/A	negative
6	brain parenchyma	right frontal lobe	1	20 × 20 × 19	CT MRI	yes	High	no	yes	no	restricted	increased	positive

LEGENDS: CT, computed tomography female; MRI, magnetic resonance imaging; MIBG, metaiodobenzylguanidine; CNS, central nervous system, CNS; N/A, not available.

**Table 3:** Main studies reporting occurrence of CNS metastasis in NB are resumed.

Observation Period	Incidence of CNS Metastasis	Reference
1980–1999	6.3%	[15]
1989–2002	8%	[16]
1965–2000	8%	[17]
1982–1989	5%	[18]
1985–2000	5.3%	[11]
1990–2007	10.8%	[28]
2002–2015	2.7%	[31]

## Data Availability

Data are available from the corresponding author on reasonable request.
